# Inhibition of JAK2/STAT3 signaling pathway by panaxadiol limits the progression of pancreatic cancer

**DOI:** 10.18632/aging.203575

**Published:** 2021-10-08

**Authors:** Xuhui Fan, Haotian Fu, Ni Xie, Hangcheng Guo, Tiantian Fu, Yunfeng Shan

**Affiliations:** 1 The First Affiliated Hospital of Wenzhou Medical University, Wenzhou 325000, China; 2 Department of Radiology, Shanghai General Hospital, Shanghai Jiao Tong University School of Medicine, Shanghai 200080, China; 3 Department of Gastroenterology, Shanghai General Hospital, Shanghai Jiao Tong University School of Medicine, Shanghai 200080, China

**Keywords:** panaxadiol, pancreatic cancer, apoptosis, proliferation, JAK2-STAT3

## Abstract

Pancreatic cancer is the fourth leading cause of cancer-related death with the characteristics of chemoresistance and early metastasis. Panaxadiol, a triterpenoid saponin extracted from the roots of American ginseng, has been proved to display anti-tumor activity in colon cancer. In this study, we found panaxadiol significantly inhibited proliferation, and induced apoptosis in human pancreatic cancer cell lines PANC-1 and Patu8988 in a dose-dependent manner. Furthermore, the expression of apoptosis-related proteins (Bax, Bcl2, Cleaved-caspase3) was detected via western blot and immunofluorescence staining. In addition, panaxadiol was also found to inhibit the migration of pancreatic cancer cells by wound healing and transwell assays. *In vivo*, the growth of xenograft pancreatic cancer models was also notably suppressed by panaxadiol compared to the control group. Moreover, the down-regulation of JAK2-STAT3 signaling pathway was responsible for the underlying pro-apoptosis mechanism of panaxadiol, and this result was in good agreement with molecular docking analysis between panaxadiol and STAT3. In conclusion, our work comprehensively explored the anti-tumor ability in PANC-1 and Patu8988 cells of panaxadiol and provided a potential choice for the clinical treatment of pancreatic cancer patients.

## INTRODUCTION

Pancreatic cancer is one of the most lethal tumors associated with its 9% five-year survival rate and ranks fourth among the leading causes of cancer-related death in the United States [1]. Surgery offers the only curable treatment for pancreatic cancer patients [2]. Unfortunately, because of a lack of obvious symptoms in the early stage and a high possibility of metastasis, about 80~85% of patients lose the opportunity for surgery when first diagnosed at the advanced stage [3, 4]. For those individuals, treatment with chemotherapy drugs has been the mainstream choice, such as gemcitabine, which plays an essential role as the first-line chemotherapy agent [5]. Nevertheless, chemoresistance is another problem among current chemotherapy agents for pancreatic cancer, including gemcitabine. Accordingly, discovering an effective drug to provide an alternative option is urgently needed.

**Figure 1:**
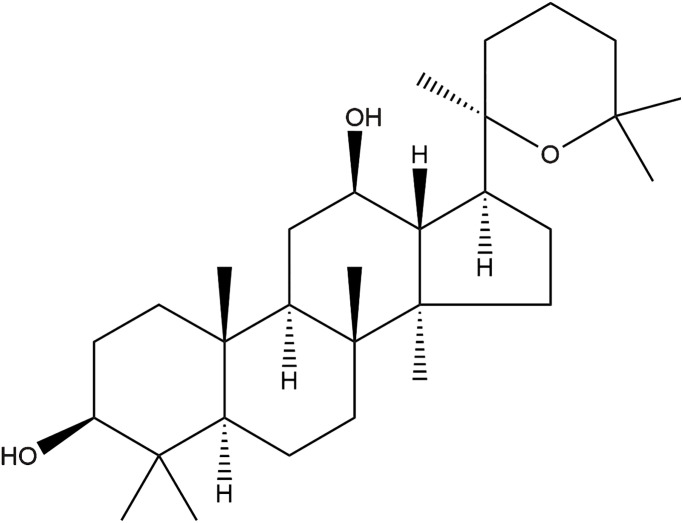
The chemical structure of panaxadiol.

Recently, natural products are gaining increasing attention for their potential anti-cancer function. For example, some constituents of ginseng have demonstrated excellent therapeutic efficacy and favorable safety for the treatment of cancer [6]. Panaxadiol (Figure 1), extracted from the roots of American ginseng, is a triterpenoid sapogenin monomer with a dammarane skeleton. It exhibits multiple biological activities, including anti-cancer [7–9], anti-inflammatory [10] and radioprotective effects [11]. Wang et al. showed panaxadiol could be used to treat colon cancer via suppressing the expression of PD-L1 and tumor proliferation [7]. However, whether panaxadiol plays a role in pancreatic cancer treatment has not been investigated.

In this study, we researched the anti-proliferation, pro-apoptosis, and anti-metastasis effects of panaxadiol on human pancreatic cancer, and we revealed the potential molecular mechanisms. Accordingly, this study helped to offer a novel alternative agent for pancreatic cancer treatment and further clarified the anti-tumor mechanisms of panaxadiol based on the previous researches [6–9].

## RESULTS

### Panaxadiol suppressed the proliferation of human pancreatic cancer cells

**Figure 2:**
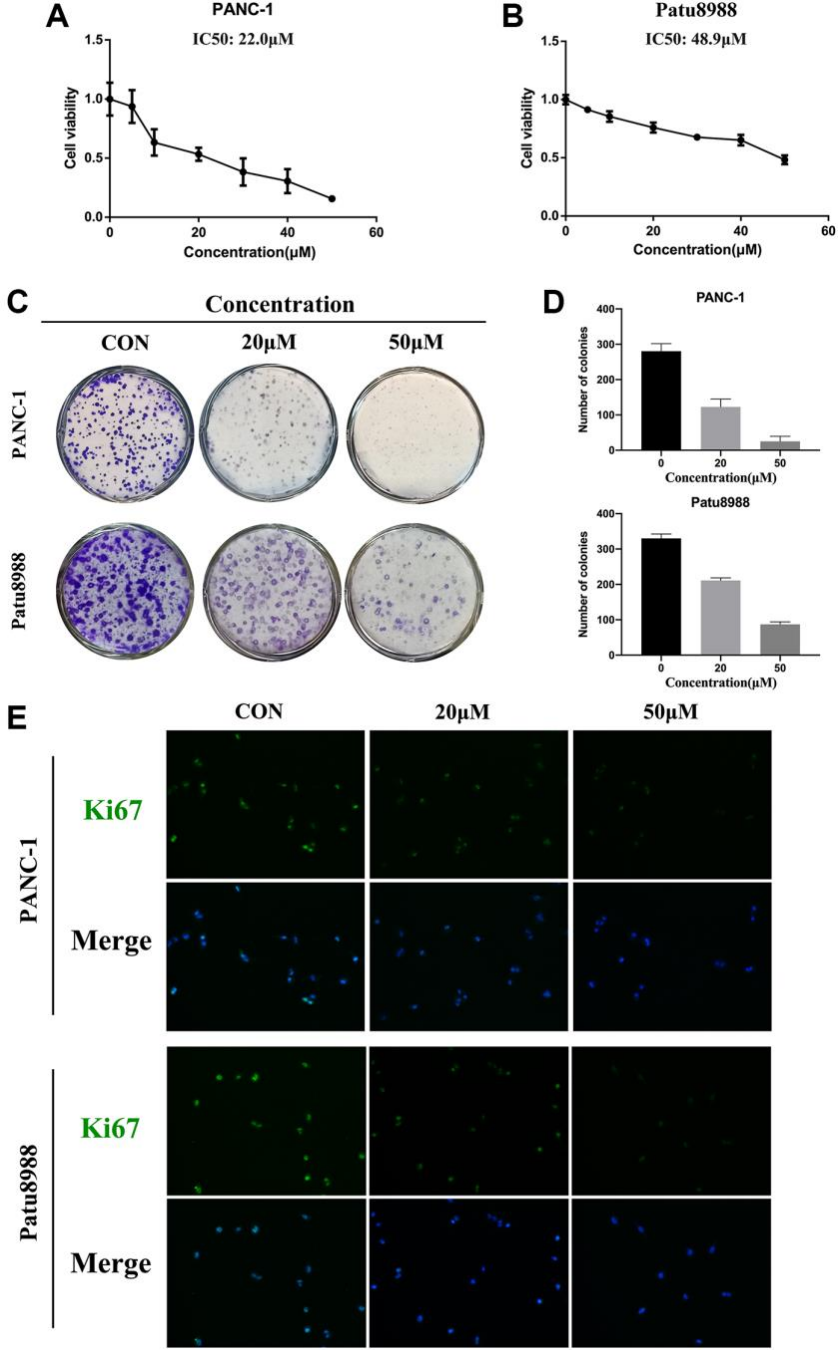
**Panaxadiol suppressed the proliferation of human pancreatic cancer cells.** (**A**–**B**) The cell viability of PANC-1 and Patu8988 was detected by CCK8 assays. The IC50 values of panaxadiol for PANC-1 and Patu8988 cells were 22.0 μM and 48.9 μM correspondingly. PANC-1 and Patu8988 were treated with different concentrations of panaxadiol as soon as they were seeded in 96-well plates, and then they were incubated for 24 h. (**C**–**D**) Colony formation assays showed the proliferation of pancreatic cancer cells was suppressed. When the cells were plated in a 6-well plate and incubated for 24 h, 0, 20 μM, and 50 μM of panaxadiol was added to each well. After 14 days, the cells were fixed and stained with crystal purple to count the number of colonies. The results were presented as mean ± SD; ^*^P < 0.05. (**E**) Ki67 immunofluorescence staining showed the Ki67 positive cells decreased after the treatment of panaxadiol.

Firstly, CCK8 assay was conducted to detect the anti-proliferation effect of panaxadiol in pancreatic cancer cells and normal cells. As shown in Figure 2A and 2B, after the cells were treated with panaxadiol as soon as they were seeded in 96-well plates and incubated for 24 h, the viability of PANC-1 and Patu8988 cells was decreased in a dose-dependent manner compared to control groups, and the IC50 values of panaxadiol for PANC-1 and Patu8988 cells were 22.0 μM and 48.9 μM correspondingly. Meanwhile, panaxadiol had no obvious toxicity on normal human HPNE cell line (Supplementary Figure 1). Based on the result of CCK8, we chose 20 μM and 50 μM as the experimental concentrations. As colony formation assay (Figure 2C and 2D) demonstrated, the number of colonies of both PANC-1 and Patu8988 cells treated with panaxadiol significantly decreased in comparison to control groups (*P* < 0.05). Furthermore, Ki67 could be an indicator of proliferation [12] and it was highly expressed in pancreatic tissues as documented [13]. In our study, immunofluorescence staining (Figure 2E) showed that the rate of Ki67 positive cells in experimental groups was obviously less than control groups. To sum up, the experimental results mentioned above confirmed that panaxadiol could suppress the proliferation of pancreatic cancer cell lines significantly.

### Panaxadiol induced apoptosis in PANC-1 and Patu8988 cells

**Figure 3:**
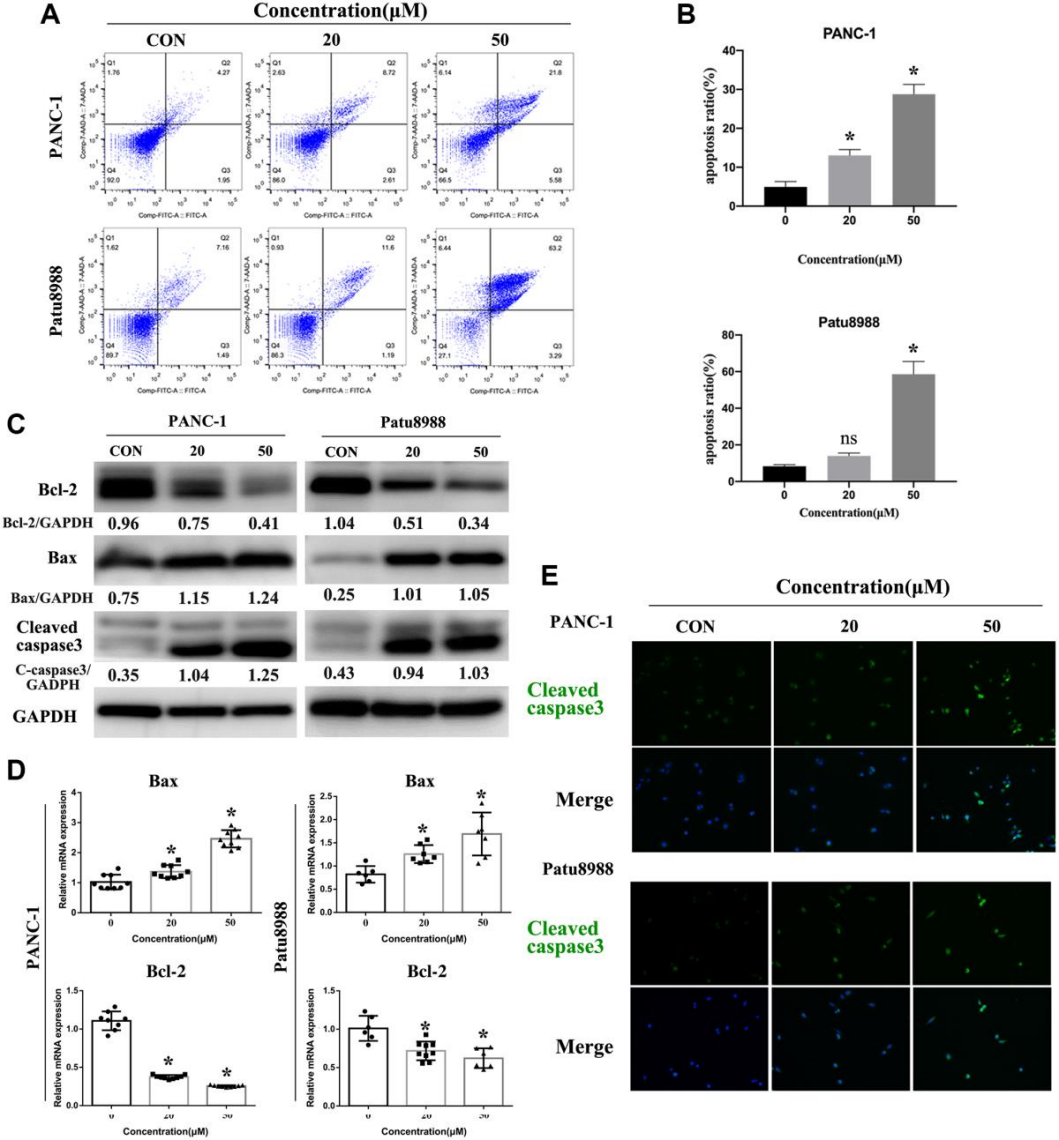
**Panaxadiol induced apoptosis in PANC-1 and Patu8988 cells.** (**A**–**B**) Flow cytometry of apoptosis demonstrated apoptosis ratio of pancreatic cancer cells was significantly increased after being incubated with different concentrations of panaxadiol, and apoptosis ratio = (Q2+Q3)/(Q1+Q2+Q3+Q4), ^*^P < 0.05. (**C**) The expression of apoptosis-related proteins (Bcl-2, Bax, Cleaved caspase3) were detected by western blot. The ratios of Bcl-2/Bax of PANC-1 were 1.29, 0.64, 0.32 as the concentration of panaxadiol increased gradually. For Patu8988, the ratios were 4.04, 0.51, 0.33 accordingly. (**D**) qRT-PCR confirmed the same results as western blot from the expression of gene level. (**E**) Cleaved caspase3 was up-regulated in PANC-1 and Patu8988 cells after being treated with panaxadiol by performing immunofluorescence staining.

In consideration of panaxadiol inhibiting the proliferation of cells, we tried to identify the pro-apoptosis effect of panaxadiol, and flow cytometry assay was performed after PANC-1 and Patu8988 cells being treated with 0, 20 μM, 50 μM panaxadiol for 24 h. AnnexinV-FITC/7-AAD staining (Figure 3A and 3B) quantified the ratio of early and late apoptosis cells. The results demonstrated apoptosis ratio of experimental groups was notably higher than control groups (*P* < 0.05), especially for Patu8988 cells (the mean apoptosis ratio of 50 μM groups achieved 58.58%). Furthermore, we explore the changes of apoptosis-related proteins expression (Bax, Bcl-2, Cleaved-caspase3) using western bolt, qRT-PCR and immunofluorescence staining. As shown in Figure 3C, the expression of mitochondrial apoptosis related protein Bax and pro-apoptosis marker Cleaved-caspase3 was up-regulated, while anti-apoptotic related protein Bcl-2 was down-regulated. Furthermore, the ratio of Bcl-2/Bax of PANC-1 were 1.29, 0.64, 0.32 when the administration concentration of panaxadiol increased, which explained the phenotype of apoptosis that our experiments demonstrated. For Patu8988, the ratios were 4.04, 0.51, 0.33 accordingly. Besides, the same changes of Bax and Bcl-2 also got confirmed (*P* < 0.05) at the mRNA level by qRT-PCR (Figure 3D). In addition, the ratio of Cleaved-caspase3 positive cells was visibly higher in panaxadiol treated groups by immunofluorescence staining (Figure 3E).

### Panaxadiol inhibited metastasis of human pancreatic cancer cells

**Figure 4:**
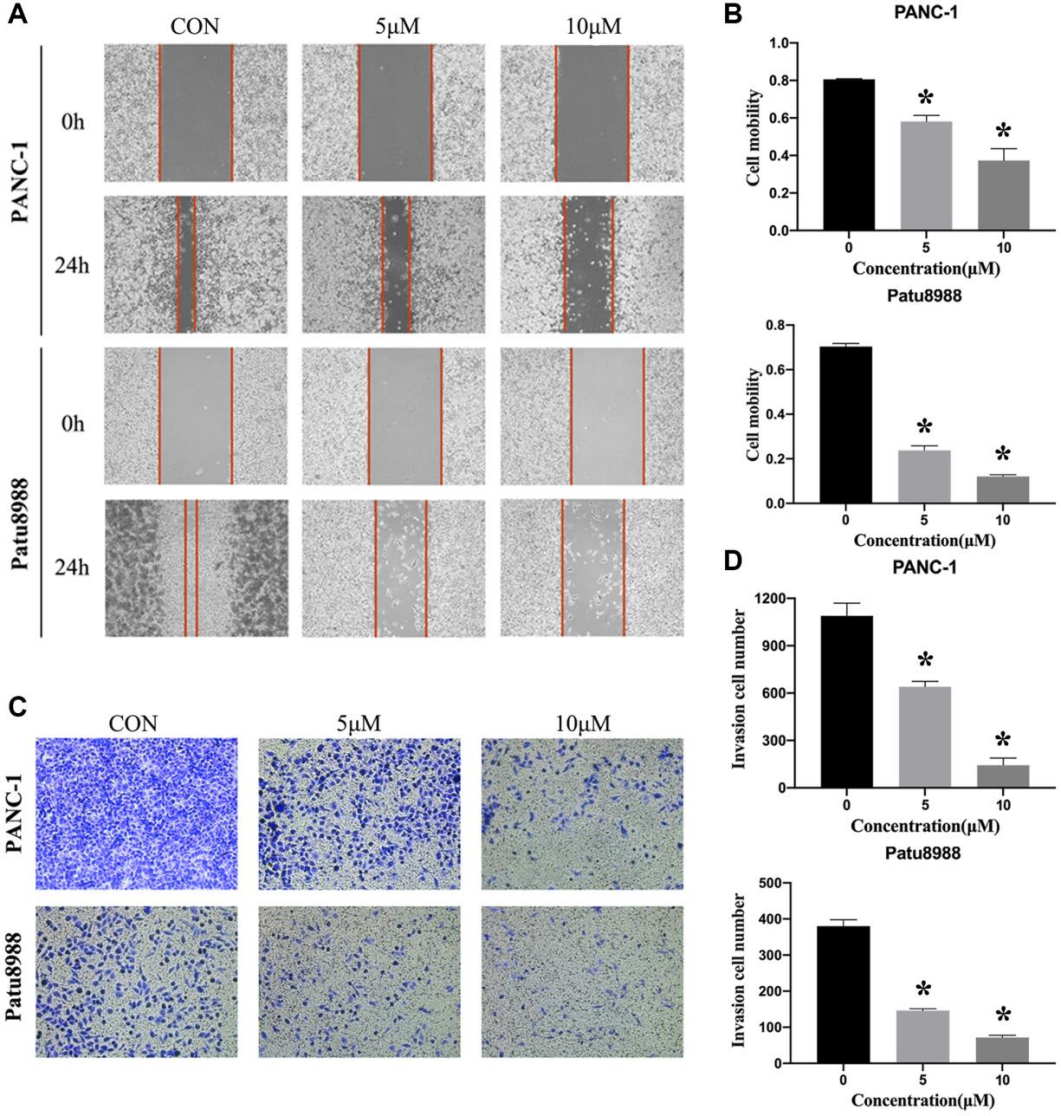
**Panaxadiol inhibited the migration of human pancreatic cancer cells.** (**A**–**B**) Wound healing assay demonstrated the migration ability of PANC and Patu8988 was inhibited by panaxadiol in a dose-dependent manner. Meanwhile, we also used serum-free medium to exclude the effect of cell proliferation on the migration experiments. (**C**–**D**) Transwell assay proved panaxadiol suppressed the migration of PANC-1 and Patu8988 cells compared to control groups after being incubated with 0, 5 μM, 10 μM panaxadiol for 24 h. ^*^P < 0.05.

In addition to the function in anti-proliferation and pro-apoptosis, we were interested in determining whether panaxadiol could inhibit metastasis of PANC-1 and Patu8988 as well. As shown in Figure 4A and 4B, the cell mobility was significantly decreased when the concentration of panaxadiol increased (*P* < 0.05). What’s more, transwell assay also revealed similar results, the number of migration cells was decreased in a dose-dependent manner (*P* < 0.05, Figure 4C and 4D).

### Panaxadiol exerted its pro-apoptotic effect via JAK2-STAT3 signaling pathway

**Figure 5:**
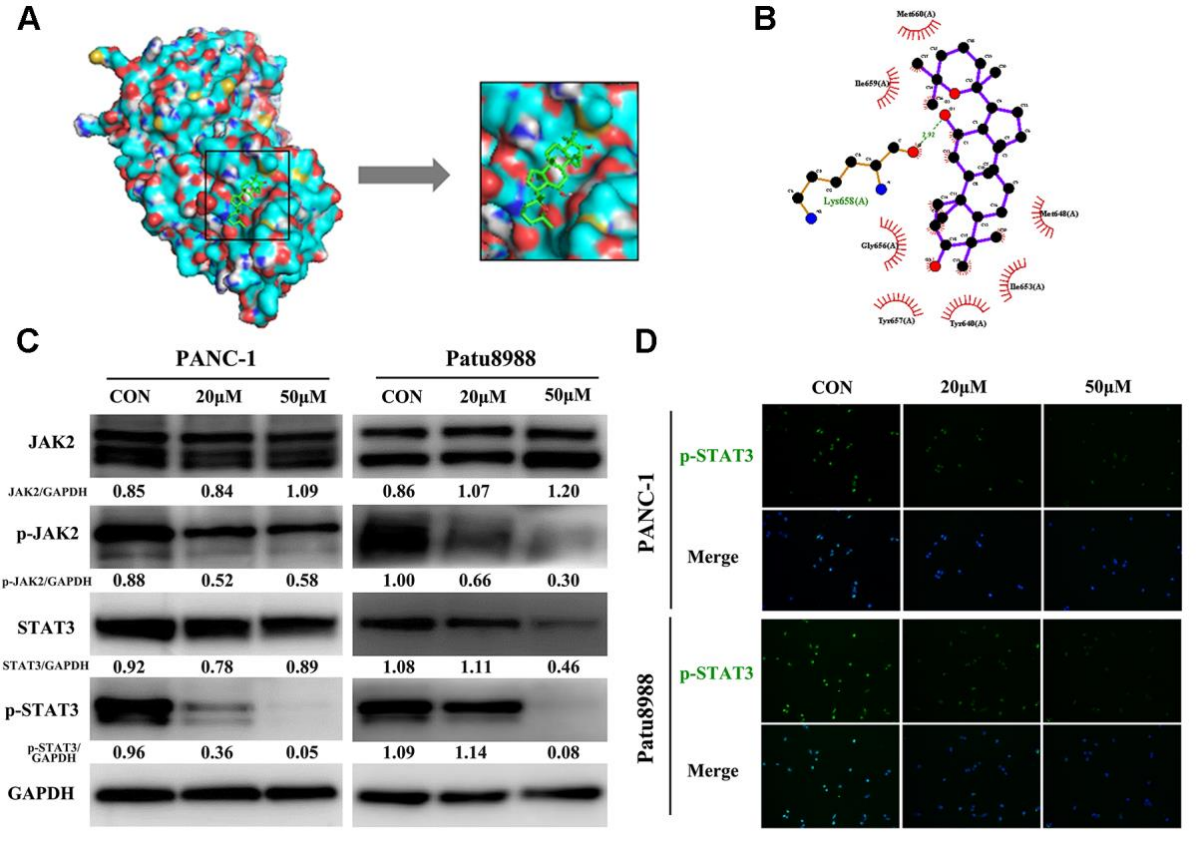
**Panaxadiol exerted its pro-apoptotic effect via JAK2-STAT3 signaling pathway.** (**A**) Panaxadiol bound to the binding domain on STAT3 distinctly in molecular docking analysis, the right image was the magnified picture from the left. The lowest binding energy with the target site was −5.7 (kcal/mol) among them (**B**) The surface view of docking analysis in 2D. The corresponding predicted binding site of STAT3 to panaxadiol was Src Homology 2 domain (SH2) (**C**) The protein expression of JAK2-STAT3 signaling pathway after being incubated with panaxadiol for 24 h. (**D**) Panaxadiol decreased the ratio of p-STAT3-positive cells in pancreatic cancer cells.

To explore the underlying pro-apoptosis mechanism of panaxadiol, we performed molecular docking and western blot. According to the literature, blockage of JAK2-STAT3 signaling pathway was associated with apoptosis of pancreatic cancer [14, 15], and the high expression of JAK2 in pancreatic cancer patients predicts an unfavorable prognosis in most cases [16]. In molecular docking analysis (Figure 5A and 5B), panaxadiol shows promising affinity with STAT3 and the lowest binding energy which means the best binding affinity with the target site was −5.7 (kcal/mol) among them. Panaxadiol makes hydrogen bonds (green dotted line) with Lys658, and it also creates a good group of a hydrophobic pocket (red symbol) formed by Met648, Ile653, Tyr640, Tyr657, Gly656, Ile659 and Met660. The corresponding predicted binding site of STAT3 to panaxadiol was Src Homology 2 domain (SH2, residues 586–688) [17], which prevented the tyrosine phosphorylation, dimerization and nuclear translocation of STAT3, and consequently induced apoptosis [18]. Moreover, the protein expression of p-JAK2 and p-STAT3 was remarkably reduced while the total expression of JAK2 and STAT3 remained unchanged in western blot analysis (Figure 5C). At the same time, immunofluorescence staining revealed that p-STAT3 expression and nucleation decreased after treatment with panaxadiol (Figure 5D).

### Panaxadiol suppressed tumor growth in xenograft pancreatic cancer models

**Figure 6:**
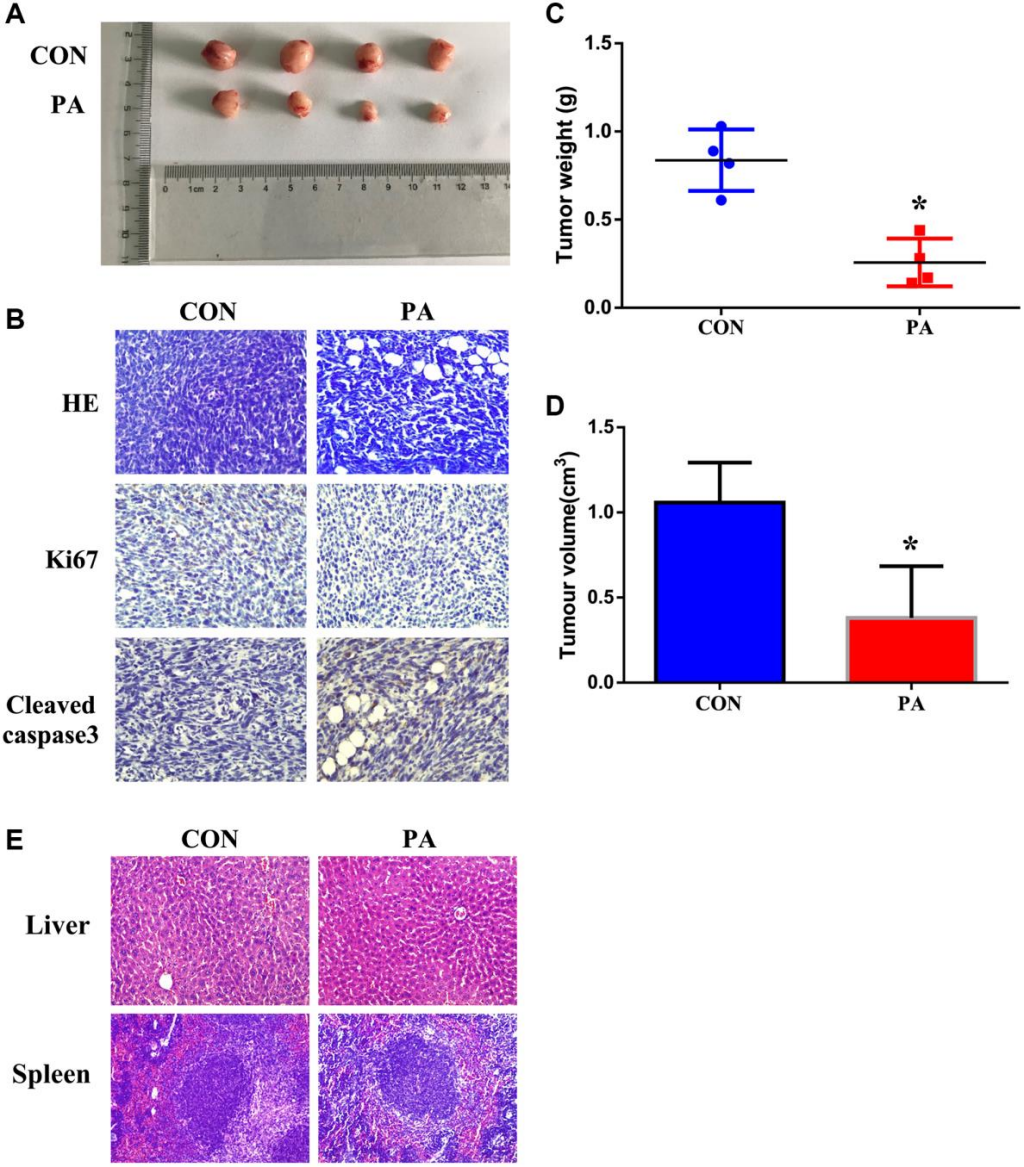
**Panaxadiol suppressed tumor growth and reduced weights in xenograft pancreatic cancer models.** (**A**) General changes of xenograft cancer models were by panaxadiol. (**B**) HE staining showed the pathological effect of panaxadiol between control and experimental groups, and immunohistochemical staining demonstrated the changes of Ki67 and Cleaved-caspase3 in tissues. (**C**–**D**) Panaxadiol significantly decreased the tumor weight and volume compared to control groups after being treated for 30 days. ^*^P < 0.05. (**E**) The HE staining of liver and spleen. *In vivo*, panaxadiol possessed almost no cytotoxicity against normal tissues.

Moreover, we also identified the anti-tumor effect of panaxadiol *in vivo*. When compared to the control group, the tumor weight and volume of panaxadiol -treated group were notably lower (*P* < 0.05, Figure 6A, 6C and 6D). Furthermore, immunohistochemical staining exhibited the expression of Ki67 was increased while Cleaved-caspase3 was decreased (Figure 6B), which were identical to the results of experiments *in vitro*. In addition, the HE staining of liver and spleen demonstrated that panaxadiol possessed almost no cytotoxicity against normal tissues (Figure 6E).

## DISCUSSION

Pancreatic cancer is one of the most malignant tumors with rapid progression and a high mortality rate [19]. Due to the lack of specific symptoms in the early stage, a majority of patients lost the only curable opportunity of surgery, when they were diagnosed as the advanced stage [2, 3]. For these patients, chemotherapy was the remaining efficient treatment [20]. However, pancreatic cancer is remarkably resistant to current chemotherapeutic drugs and it usually leads to the failure of the treatment. Therefore, it is urgent to develop new agents which are more effective and have fewer side effects. Nowadays, some natural products have been found to play an increasingly important role in tumor treatment [21]. Panaxadiol, a triterpenoid saponin of ginsenoside derived from American ginseng roots, has been proved to exhibit multiple pharmacological activities against several kinds of cancer [7–9, 22]. Xiao et al. found panaxadiol promoted apoptosis in human hepatocellular carcinoma cells via the mitochondrial pathway [22]. Li et al. revealed the anti-cancer capacity of 5-fluorouracil could be distinctly boosted by panaxadiol on colorectal neoplastic cells [9]. Based on the former researches, whether panaxadiol exhibits a favorable treatment effect on pancreatic cancer aroused our interest. In our current study, we have observed that panaxadiol suppressed proliferation and metastasis apparently, inducing apoptosis in human pancreatic cancer cells PANC-1 and Patu8988. In addition, panaxadiol inhibited tumor growth in xenograft pancreatic cancer models.

Apoptosis is one of the physiological processes of cells, and the balance between apoptosis and proliferation is essential to maintain cell homeostasis [23]. Apoptosis can not only remove harmful or unnecessary cells but also make a great contribution to the prevention of tumorigenesis [24]. Nevertheless, the dysregulation of apoptosis is one of the major features of cancer, which may directly result in tumor proliferation and subsequent malignant behavior. Therefore, plenty of agents exert their anti-cancer effects by promoting cell apoptosis [25]. At present, there are two known pathways of cell apoptosis: the death receptor-mediated extrinsic pathway and the mitochondria-mediated intrinsic pathway. After receiving specific internal stimulation, the intrinsic apoptosis pathway will be initiated. Afterward, pro-apoptosis proteins (Bax, Bak) will be activated and anti-apoptosis proteins (Bcl2s, Bcl-xl and Mcl-1) will be inactivated. These apoptosis-related proteins act on mitochondria together which leads to the change of mitochondrial membrane potential and permeability. Besides, apoptosis is partly determined by the ratio of anti-apoptosis/pro-apoptosis such as Bcl-2/Bax, rather than the expression of Bcl-2 or Bax alone [26]. As a result, cytochrome c, Apaf-1, caspase 9 and other initiator factors bind to allow for cleavage and activation of key apoptosis effectors (caspase 3 and 7) [27, 28]. In our study, flow cytometry analysis proved panaxadiol induced cell apoptosis of PANC-1 and Patu8988 in a dose-dependent manner, and the expression of apoptosis-related proteins (Bax, Bcl-2, Cleaved-caspase3) was also detected to confirm the corresponding mechanism of apoptosis by western blot and immunofluorescence staining.

In previous researches, JAK2-STAT3 signal pathway has been proved to play a critical role in tumorigenesis, proliferation, stroma modification, chemoresistance, immune cell infiltration, patient survival time in pancreatic cancer [16, 29, 30]. After receiving the stimulation of the upstream signal, JAK2 is activated by tyrosine phosphorylation of the receptor, and this also leads to phosphorylation of STAT3. Then, P-STAT3 forms a dimer and entry the nucleus to regulate various target genes. It was documented that the expression of JAK2 and STAT3 was particularly high in pancreatic cancer tissues compared with para-cancerous tissues and it was intensely associated with a poor prognosis in pancreatic cancer patients [16]. According to Wang et al. [7], JAK2-STAT3 signal pathway was involved in the anti-tumor effect of panaxadiol in colon cancer. Furthermore, microRNA-216a has been reported to be one of the inhibitors of JAK2-STAT3 [31], and its therapeutic mechanism and effect of treating pancreatic cancer were similar to panaxadiol, which reduced the expression of p-JAK2 and p-STAT3 of pancreatic cancer cells and block cancer progression *in vivo* and *in vitro*. In our experiments, we demonstrated the expression of p-JAK2 and p-STAT3 was reduced after the treatment of panaxadiol while the expression of total JAK2 and STAT3 remained approximately unchanged. Therefore, JAK2-STAT3 signal pathway plays a critical role in the treatment of pancreatic cancer with panaxadiol as well.

Early metastasis is one of the main reasons for the high mortality of pancreatic cancer. Metastasis of tumors is a complex process, and migration is indispensable in the initial step [32]. In our wound healing assay and transwell assay, panaxadiol significantly suppressed the migration of PANC-1 and Patu8988 in a dose-dependent manner. Hence, panaxadiol also inhibits early metastasis and improves the prognosis of pancreatic cancer patients.

In conclusion, we elucidated the anti-tumor effects of panaxadiol in inhibiting proliferation, inducing apoptosis, and suppressing migration in PANC-1 and Patu8988 cell lines. In addition, we indicated that JAK2-STAT3 signal pathway might be the relevant mechanism of action. Our work provided a novel agent for the treatment of pancreatic cancer, and many further studies of panaxadiol and how it works in clinical trials are required to be performed for the next phase of research.

## MATERIALS AND METHODS

### Drugs and antibodies

Panaxadiol powder was purchased from Yuanye Biotechnology (B21040, purity ≥ 98%, Shanghai, China) and dissolved in dimethyl sulfoxide (DMSO) at a concentration of 1 mM. The following primary antibodies were used: Bcl2 (Proteintech), Bax (Cell Signaling Technology), Cleaved caspase-3 (Proteintech), STAT3 (Abcam), P-STAT3 (Abcam), JAK2 (Abcam), P-JAK2 (Abcam), GAPDH (Bioworld Technology). The antibodies above were diluted to 1:1,000.

### Cell culture

Human pancreatic cancer cell lines PANC-1 (CRL-1469), and Patu8988 (PTA-8988) were procured from American type culture collection (Manassas, VA, USA). These two types of cells were cultured in Dulbecco’s modified Eagle’s medium (DMEM; Invitrogen, Carlsbad, CA, USA), which contained 10% fetal bovine serum (FBS; Gibco, USA) and 1% penicillin-streptomycin (Invitrogen). Both cells were maintained at 37°C under 5% CO_2_ humidified atmosphere.

### Cell viability assay

The Cell count kit-8 (CCK8; HY-K0301, MedChemExpress, Shanghai, China) assay was conducted to detect the cell proliferation and cytotoxicity according to the manufacturer’s protocol. PANC-1 and Patu8988 cells were seeded in 96-well plates (5 × 10^3^ per well) at different concentrations of panaxadiol. After 24 h, 10 μl of CCK-8 reagent was added to each well to incubate at 37°C for another 2 h in the dark. Then, the cell viability was determined by comparing the absorbance values at OD450 to the control group.

### Colony formation assay

Approximately 500 cells were added to 6-well plates with 2 ml cell culture medium. After being incubated for 24 h, 0, 20 μM, and 50 μM of panaxadiol was added to each well. When the cells were cultured for 14 days and single cell colonies were formed which could be visible by naked eyes, the cells were fixed by formaldehyde and stained with 0.5% crystal purple to count the number of colonies.

### Wound healing assay

PANC-1 and Patu8988 cells were seeded to 6-well plates and cultured to confluence. Next, a crystal pipette tip was applied to scratch a linear would and the floating cells were washed away with PBS. Then, the cells were treated under 0, 5 μM, 10 μM panaxadiol with serum-free culture medium to exclude the effect of cell proliferation. As time went by, the cells would refill the linear gaps, which were captured using a microscope every 24 h.

### Transwell assay

Transwell assay was performed to detect the cells migration ability, using transwell chambers (Costar, NY, USA). A total of 3 × 10^4^ cells in 200 ul serum-free media with indicated concentrations of panaxadiol were seeded into the upper chamber, and 600 μl DMEM contained 10% FBS was added to the lower chamber. After being incubated at 37°C for 24 h, the cells on the upper membrane were removed using cotton swabs, and the cells on the lower membrane were fixed with formaldehyde and stained with 0.5% crystal purple. Next, the invaded cells were counted in five different fields under a microscope.

### Flow cytometry analysis

The cells were treated with panaxadiol in 6-well plates for 24 h and collected by centrifugation. After being resuspended in 500 ml 1X binding buffer, the cells were incubated with 5 μl Annexin V-FITC under dark conditions for 15 min and stained with 5 ul 7-Aminoactinomycin D (7-ADD) for another 5 min. Next, Apoptosis analysis was performed using flow cytometry (BD Biosciences, USA).

### Immunofluorescence staining

The cells were added to glass slides in 6-well plates treated with different concentrations of panaxadiol. The next day, the cells were washed by PBS, fixed with 4% paraformaldehyde for 30 min, permeabilized by 0.1% Triton X100 for 15 min, and sealed in 5% normal goat serum for 1 h. After these steps, the cells were incubated overnight with primary antibodies: Ki67 (dilution ratio: 1:100; Cell Signaling), c-Caspase3 (dilution ratio: 1:100; Santa Cruz), p-STAT3 (dilution ratio: 1:100; Santa Cruz). Then, the cells were incubated with appropriate second antibodies (dilution ratio: 1:400; Santa Cruz) in dark condition for 1 h, and nuclei were stained with 4′,6-diamidino-2-phenylindole (DAPI, Invitrogen) for 5 min. At last, the cells were detected under immunofluorescence microscopy.

### Quantitative reverse transcription-polymerase chain reaction (qRT-PCR)

TRIzol reagent (TaKaRa, Japan) was used to isolate total RNA from the cell after drug action according to the manufacturer’s protocol. Then, 2 μg of total RNA was transcribed to cDNA in a 20 μl reaction mixture (TaKaRa, Japan). The qPCR was carried out using the SYBR Green Master Mix kit (TaKaRa, Japan) and specific primers (Sangon Bio, Shanghai, China) for amplification. The samples were analyzed by 7500 fast PCR system (Applied Biosystems, USA), and melting curves were used to test the primer specificity. β-actin was regarded as a housekeeping gene and normalizer. Finally, the qualitative results were calculated using the ΔΔCT method. The primer sequences (5′–3′) used in this study were listed as follows: β-actin: F: TGACGTGGACATCCGCAAAG; R: CTGGAAGGTGGACAGCGAGG; Bax: F: TTTCTGACGGCAACTTCAACTG; R: CGGAGGAAGTCCAATGTCCAG; Bcl-2: F: CTTCGCCGAGATGTCCAGC; R: CCAGTTCACCCCGTCCCT.

### Western blot analysis

After treatment with different concentrations of panaxadiol for 24 h, the cells were lysed in RIPA lysis buffer (Beyotime, China) for 30 min on ice. The total protein concentration was measured using the BCA method (Beyotime, China). After being mixed with 5X loading buffer and being boiled for 5 min, the total protein was separated by 12% SDS-PAGE and transferred to PVDF membranes (Solarbio, China). Then, 5% skim milk in TBST was used to block the blots and the membranes were incubated with primary antibodies (dilution ratio: 1:1,000) at 4°C overnight. Next, membranes were washed by TBST and incubated with secondary antibodies (dilution ratio: 1:5,000) for 1 h. In the end, the bands were visualized via chemiluminescence with autoradiographic film (Thermo Scientific, USA).

### Molecular docking

The STAT3 protein downloaded from PDB website and panaxadiol were chosen to perform the molecular docking simulations. The software AutoDockTools 1.5.6 was used to predict the best binding site of panaxadiol and STAT3 with the highest affinity as well as the hydrogen-bonding interactions.

### Nude mouse tumorigenicity assay

The animal procedures were adhered to regulatory institutional guidelines for animal welfare (NIH Publications, No.80–23), and were examined and approved by the Institutional Animal Care and Use Committee of Wenzhou Medical University, China. 6 weeks old BALB/c nude mice weighing about 18–22 g were acquired from Wenzhou Medical University Experimental Animal Centre. The mice were reared under the same conditions, such as humidity and temperature, and fed with the standard chow and water. 5 × 10^6^ PANC-1 cells suspended in 100 ul PBS were subcutaneously injected into the right leg of mice, and the mice fasted the night before injection. As for the experimental group, panaxadiol (25 mg/kg) was administrated intraperitoneally every 3 days for 30 days. In the end, the mice were euthanized by overdose CO_2_. The tumor volume was measured based on the formula: V = (length × width^2^)/2, and the length referred to the longest dimension.

### Histopathological analysis and immunohistochemistry staining (IHC)

Formalin-fixed and paraffin-embedded tumor samples were cut into sections (4 μm) and stained with hematoxylin and eosin (HE, Yuanye Bio, China). As for immunohistochemistry analysis, the sections were dewaxed in xylene and rehydrated in ethanol gradually. Afterward, 0.1% sodium citrate was used for antigen retrieval, and 3% H_2_O_2_ was used to block endogenous peroxidase along with 5% normal goat serum to block non-specific sites. After these steps, the sections were incubated in primary antibodies (Ki67 and Cleaved-caspase3, dilution ratio: 1:200, Abcam) overnight. At last, IHC was conducted via the streptavidin-peroxidase method. All images were obtained by digital microscope (Leica Microsystems, Germany).

### Statistical analysis

The statistical analysis in this study is performed using GraphPad Prism 9.0. The date analysis was expressed as mean ± SD. One-way ANOVA was used to determine the significant difference between experimental groups vs. control groups. Comparisons were indicated to have statistical significance if *P* < 0.05.

## Supplementary Materials

Supplementary Figure 1
