# Supplementary material for: Inhibition of JAK2/STAT3 signaling pathway by panaxadiol limits the progression of pancreatic cancer
Source: Aging (Albany NY). 2021 Oct 8;13(19):22830–42. doi: 10.18632/aging.203575 (PMC8544303; doi:10.18632/aging.203575)
Supplement: Supplementary Figure 1 [file aging-13-203575-s001.pdf]

SUPPLEMENTARY FIGURE

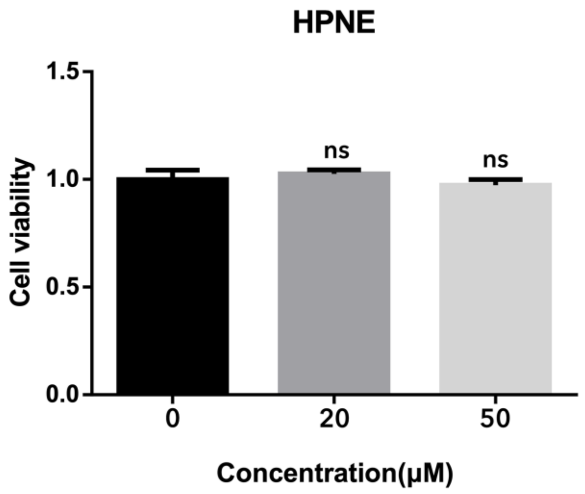

Supplementary Figure 1. Panaxadiol did no obvious effect on human normal cells HPNE. After being incubated with panaxadiol for 24 h, the cell viability of HPNE was not altered significantly. ns, $P < 0.05$.
